# Home-based enzyme replacement therapy in children and adults with Pompe disease; a prospective study

**DOI:** 10.1186/s13023-023-02715-4

**Published:** 2023-05-08

**Authors:** Imke A. M. Ditters, Nadine A. M. E. van der Beek, Esther Brusse, Ans T. van der Ploeg, Johanna M. P. van den Hout, Hidde H. Huidekoper

**Affiliations:** 1 Department of Pediatrics, Center for Lysosomal and Metabolic Diseases, Erasmus MC University Medical Center, Sophia Children’s Hospital, PO Box 2060, 3000 CB Rotterdam, The Netherlands; 2 Department of Neurology, Center for Lysosomal and Metabolic Diseases, Erasmus MC University Medical Center, Rotterdam, The Netherlands

**Keywords:** Pompe disease, Glycogen storage disease type II, Enzyme replacement therapy (ERT), Alglucosidase alfa, Home treatment, Infusion associated reactions (IARs)

## Abstract

**Background:**

Pompe disease is a lysosomal storage disease treated with life-long enzyme replacement therapy (ERT). Home-based ERT has been provided in the Netherlands since 2008 because it diminishes the burden of treatment, increases patient flexibility and autonomy, and is thus a more patient-centred approach to ERT.

**Methods:**

All Dutch Pompe patients receiving alglucosidase alfa infusions at home were approached to participate in a questionnaire to validate the safety of home-based ERT. Prospective data on symptoms occurring during or within 48 h after infusion and retrospective data on infusion associated reactions (IARs) in the last three months were collected four times during one year.

**Results:**

In total, 116 out of 120 eligible patients (17 classic infantile, 2 atypical infantile, 15 childhood onset and 82 adult) filled out 423 questionnaires (response rate: 88.1%). Symptoms during or after infusion were reported 27 times in 17 patients. Fatigue was the most commonly reported health complaint (in 9.5% of patients). Four health complaints were judged to be IARs and reported to the Erasmus MC University Medical Center. None of the IARs reported in this study warranted emergency clinical care.

**Conclusions:**

Our data demonstrate that home-based ERT in Pompe disease can be safely implemented as few, mostly mild, symptoms were reported during or after infusion. Insights from this study can be used as a base for implementing home-based ERT in other countries and to further optimize patient care, as unreported mild symptoms do not pose a health risk but may still be relevant to the patient.

**Supplementary Information:**

The online version contains supplementary material available at 10.1186/s13023-023-02715-4.

## Introduction

Pompe disease, or glycogen-storage disease type 2 (OMIM #232300), is a progressive lysosomal myopathy. The disease is caused by a deficiency of the enzyme acid α-glucosidase (GAA; EC number 3.2.1.20). A differentiation is made between the classic infantile form, in which patients develop a severe cardiomyopathy and respiratory insufficiency and die before the age of 1 year without treatment, and non-classic or late-onset Pompe disease, which can be divided into atypical, childhood onset and adult Pompe disease. In the late-onset forms, patients present with an axial and limb-girdle pattern of muscle weakness and weakness of the respiratory muscles at any age but do not have a hypertrophic cardiomyopathy. An exception to this are atypical infantile patients, which do have a hypertrophic cardiomyopathy but are more mildly affected than their classic infantile counterparts [1–5]. Since 2006, enzyme replacement therapy (ERT) with alglucosidase alfa (Myozyme) has been available [1]. In classic-infantile Pompe patients, ERT prolongs lifespan, enables patients to reach previously unmet motor milestones, improves motor outcomes and ventilator-free survival, and generally leads to normalisation of the left ventricular mass index (LMVI) in the majority of classic-infantile patients [6–10]. Notably, classic infantile patients can nowadays reach adulthood due to treatment with ERT. In late-onset patients, ERT improves or stabilises skeletal muscle strength, muscle function, and respiratory function; in addition, survival is improved [11–19].

Since 2008 home-based ERT has been provided for Pompe patients in the Netherlands, as this diminishes the burden of treatment, allows for more patient flexibility and autonomy [20] and is thus a more patient-centred approach to ERT. Over 80% of Dutch Pompe patients receive their infusions at home. However, in most countries, ERT is only provided in hospital due to the risk of severe infusion associated reactions (IARs) reported on the label, such as anaphylaxis, severe allergic and immune-mediated reactions and risk of cardiorespiratory failure [21, 22].

We recently demonstrated that home-based ERT can be safely provided in adults with Pompe disease [23] based on the analysis of 18,380 infusions. In the present study, we further investigated the safety of home-based therapy by conducting a prospective survey over one year in children and adults, covering the whole Pompe disease spectrum. We included patients with the classic infantile, atypical infantile, childhood onset and adult phenotypes. Patients were asked about complaints during infusion and the 24 to 48 h afterwards, as in the literature, IARs are also reported in the 48 h after alglucosidase alfa administration [24]. Additionally, we assessed the ability of our current protocol to capture minor health complaints, to further optimise care with home-based infusions for Pompe disease if needed.

## Methods

### Study population

All Dutch patients with Pompe disease, across the whole disease spectrum (classic infantile, atypical infantile, childhood onset and adult [1–5]), currently receiving home-based ERT with alglucosidase alfa, were approached to participate in the study. All patients had a confirmed diagnosis of Pompe disease, as demonstrated by a deficiency of acid alpha-glucosidase (EC 3.2.1.20) in leucocytes, fibroblasts and/or two disease-causing *GAA* variants in trans.

Patients were eligible for home-based infusions after one year of ERT in the hospital if ERT was proven safe and no recent IARs had occurred [25]. Specially trained nurses administered home-based infusions of alglucosidase alfa according to a strict protocol. When patients were sick or ill prior to infusion, the hospital had to be contacted to assess whether the infusion could be administered safely. If IARs occurred, they were managed according to the standard operating procedure (SOP), and an on-call specialist team was available for consultation if needed. Standard treatment regimens in the Netherlands are 40 mg/kg/week for classic infantile patients and atypical infantile patients and 20 mg/kg/every other week for all late-onset patients [10]. Four patients in the childhood-onset group received a dosage other than the standard recommended dosage of ERT.

### Data collection

The study was reviewed by the institutional review board and was assessed as not to be subject to the Medical Research Involving Human Subjects Act (WMO). All patients or parents/legal guardians of patients receiving ERT in the home situation were approached to fill in a questionnaire four times during one year at months 3, 6, 9 and 12. Data were collected between 2021-01-08 and 2021-10-29. Questionnaires were built using Limesurvey. Gemstracker, a GCP-compliant software package, was used to undertake questionnaires and data management [26].

General demographic and treatment data were available for all patients. A core data set was collected, including data on infusions and health complaints during and 24–48 h after the infusion. Depending on answers given related to the occurrence of health complaints, the questionnaire contained five to fourteen items (see Additional files 1 and 2). The questionnaire consisted of two parts: part one included questions on the patient’s health in the week/two weeks (depending on ERT frequency) between infusions and the patient’s health during and within 48 h after the infusion was administered, with a focus on minor health complaints. The second part investigated possible IARs, the frequency of (possible) IARs and the actions taken in response to IARs in the last three months (multiple response options possible).

### Quality control

As a quality control measure, all infusions in which patients reported health complaints within 48 h post-infusion were checked to see if these complaints had also been reported to the treating hospital, the Erasmus MC University Medical Center. To do so, the patient’s hospital records of the day of infusion and the two days afterwards were checked. This was done to identify whether there were patients who potentially experienced (minor) symptoms during or shortly after receiving ERT which were not perceived as IARs (i.e. establish if there is underreporting of IARs), and to assess the reliability of the current protocol for reporting IARs occurring during home infusion of alglucosidase alfa in patients with Pompe disease.

### Statistical analysis

Statistical analysis was executed using SPSS 28.0.1.0. Data were cleaned by checking for outliers. Descriptive analyses were performed by tabulating demographic and questionnaire answers of patients.

## Results

### Study population

One hundred twenty patients were eligible for inclusion. In total, 116 of these patients (17 classic infantile, 2 atypical infantile, 15 childhood onset and 82 adult) filled in 423 questionnaires during one year. The overall response rate was 88.1% (84.7% in the classic infantile group, 100% in the atypical infantile group, 68.8% in the childhood-onset group and 92.3% in the adult group). Three questionnaires, two in the classic infantile and one in the adult group, were not fully completed; the missing answers have been indicated as such. The overall median duration of treatment with ERT was 11.3 years (range 1.0–21.9), 9.7 years (range 1.0–21.9) in the classic infantile group, 4.8 years (range 4.2–5.4) in the atypical infantile group, 10.0 years (range 1.3–21.3) in the childhood-onset group, and 12.2 years (range 1.2–15.0 in the adult group (Table 1).

**Table 1:** Characteristics of the study population

	Total	Classic infantile	Atypical infantile	Childhood-onset	Adult
Number of patients included, n	120	18	2	16	84
Number of patients who answered questionnaires, n (%) of total	116 (96.7%)	17 (94.4%)	2 (100.0%)	15 (93.8%)	82 (97.6%)
Number of questionnaires answered, n (%)	423 (88.1%)	61 (84.7%)	8 (100.0%)	44 (68.8%)	310 (92.3%)
Median age, years (range, IQR)	51.8 (1.3–84.5, 22.5–65.9)	10.0 (1.3–22.5, 7.0–13.6)	7.2 (5.6–8.8, 5.6-N.A.^*^)	18.9 (5.3–50.4, 12.4–28.2)	58.8 (34.0–84.5, 50.9–67.8)
Median age at start ERT, years (range, IQR)	41.7 (0.0–72.9, 11.2–54.4)	0.3 (0.0–0.7, 0.2–0.4)	2.4 (1.4–3.4, 1.4-N.A.^*^)	10.5 (1.7–32.3, 4.0–12.9)	50.1 (24.2–72.9, 39.8–59.4)
Median duration of ERT, years (range, IQR)	11.3 (1.0–21.9, 6.6–13.4)	9.7 (1.0–21.9, 6.7–13.5)	4.8 (4.2–5.4, 4.2-N.A.^*^)	10.0 (1.3–21.3, 3.9–15.7)	12.2 (1.2–15.0, 7.9–13.4)
Gender, n (%)					
Male	53 (45.7%)	8 (47.1%)	2 (100.0%)	9 (60.0%)	34 (41.5%)
Female	63 (54.3%)	9 (52.9%)	0 (0.0%)	6 (40.0%)	48 (58.5%)

N.A. = Not applicable ^*^ The third quartile of the IQR could not be calculated because of the small number of participants in this group

### Health complaints before, during and after infusion

The first part of our questionnaire consisted of questions about the previous infusion; to assess the ability of the current protocol to capture minor health complaints. Health complaints during or within 48 h from infusion were reported 27 times in 17 patients (6 classic infantile, 1 childhood onset, 10 adult). The majority of patients (n = 99, 85.3%) did not experience any health complaints related to ERT. Before infusion, most patients (91.8% classic infantile, 100% atypical infantile, 100% childhood onset, and 96.8% adult) did not report being sick or ill. We asked this to assess whether patients who were sick or ill before infusion were more likely to develop additional health complaints during or after infusion. Of the 13 questionnaires on which patients indicated being sick or ill before infusion, health complaints during or after infusion were reported on 5 questionnaires, versus 22 of the 410 questionnaires on which patients had not indicated being sick or ill before infusion. For an overview of health complaints during and after the last infusion, see Table 2. For a more detailed overview, see Additional file 3.

**Table 2:** Health complaints during and after the last infusion

	Total	Classic infantile	Atypical infantile	Childhood-onset	Adult
Health complaints during and or within 48 h after last infusion, n (% of questionnaires)					
Yes	27 (6.4%)	13 (21.3%)	0 (0.0%)	1 (2.3%)	13 (4.2%)
No	395 (93.4%)	47 (77.0%)	8 (100.0%)	43 (97.7%)	297 (95.8%)
Missing	1 (0.2%)	1 (1.6%)	0 (0.0%)	0 (0.0%)	0 (0.0%)
Health complaints during last infusion, n (% of questionnaires)					
Yes	18 (4.3%)	7 (11.5%)	0 (0.0%)	1 (2.3%)	10 (3.2%)
No	404 (95.5%)	53 (86.9%)	8 (100.0%)	43 (97.7%)	300 (96.8%)
Missing	1 (0.2%)	1 (1.6%)	0 (0.0%)	0 (0.0%)	0 (0.0%)
Health complaints within the first 24 h after last infusion, n (% of questionnaires)					
Yes	23 (5.4%)	11 (18.0%)	0 (0.0%)	0 (0.0%)	12 (3.9%)
No	399 (94.3%)	49 (80.3%)	8 (100.0%)	44 (100.0%)	298 (96.1%)
Missing	1 (0.2%)	1 (1.6%)	0 (0.0%)	0 (0.0%)	0 (0.0%)
Health complaints within 24–48 h after last infusion, n (% of questionnaires)					
Yes	15 (3.5%)	7 (11.5%)	0 (0.0%)	0 (0.0%)	8 (2.6%)
No	407 (96.2%)	53 (86.9%)	8 (100.0%)	44 (100.0%)	302 (97.4%)
Missing	1 (0.2%)	1 (1.6%)	0 (0.0%)	0 (0.0%)	0 (0.0%)
Complaints during last infusion*, n (% of complaints within group)					
Fatigue	12 (18.2%)	4 (12.9%)	N.A	1 (100.0%)	7 (20.6%)
Headache	6 (9.1%)	2 (6.5%)	N.A	0 (0.0%)	4 (11.8%)
Generally unwell	6 (9.1%)	3 (9.7%)	N.A	0 (0.0%)	3 (8.8%)
Chills	4 (6.1%)	2 (6.5%)	N.A	0 (0.0%)	2 (5.9%)
Other	4 (6.1%)	1 (3.2%)	N.A	0 (0.0%)	3 (8.8%)
Complaints within the first 24 h after infusion*, n (% of complaints within group)					
Fatigue	15 (19.0%)	8 (19.0%)	N.A	N.A	7 (18.9%)
Headache	12 (15.2%)	6 (14.3%)	N.A	N.A	6 (16.2%)
Generally unwell	7 (8.9%)	5 (11.9%)	N.A	N.A	2 (5.4%)
Diarrhoea	4 (5.1%)	2 (4.8%)	N.A	N.A	2 (5.4%)
Complaints within 24–48 h after infusion*, (% of complaints within group)					
Fatigue	9 (16.4%)	5 (16.1%)	N.A	N.A	4 (16.7%)
Headache	6 (10.9%)	3 (9.7%)	N.A	N.A	3 (12.5%)
Symptoms related to infusion*, n (% of questionnaires)					
Yes	15 (55.6%)	9 (69.2%)	N.A	0 (0.0%)	6 (46.2%)
No	4 (14.8%)	3 (23.1%)	N.A	0 (0.0%)	1 (7.7%)
Maybe	8 (29.6%)	1 (7.7%)	N.A	1 (100.0%)	6 (46.2%)
Hospitalised/emergency room/GP the week or two weeks after last infusion, n (% of questionnaires)					
No	410 (96.9%)	58 (95.1%)	8 (100.0%)	42 (95.5%)	302 (97.4%)
Yes	13 (3.1%)	3 (4.9%)	0 (0.0%)	2 (4.5%)	8 (2.6%)
Broken leg	3(0.7%)	1 (1.6%)	0(0.0%)	0(0.0%)	2 (0.6%)
Itching skin, nausea, headache, tingling sensation mouth	1(0.2%)	1 (1.6%)	0 (0.0%)	0 (0.0%)	0 (0.0%)
Pulmonary infection	1 (0.2%)	1(1.6%)	0 (0.0%)	0 (0.0%)	0 (0.0%)
Burn	2 (0.5%)	0 (0.0%)	0 (0.0%)	2 (4.5%)	0 (0.0%)
Inflamed intestine	1 (0.2%)	0 (0.0%)	0 (0.0%)	0 (0.0%)	1 (0.3%)
Cerebral haemorrhage	1 (0.2%)	0 (0.0%)	0 (0.0%)	0 (0.0%)	1 (0.3%)
Traffic accident	1 (0.2%)	0 (0.0%)	0 (0.0%)	0 (0.0%)	1 (0.3%)
Kidney stones	2 (0.5%)	0 (0.0%)	0 (0.0%)	0 (0.0%)	2 (0.3%)
Chest pain	1 (0.2%)	0 (0.0%)	0 (0.0%)	0 (0.0%)	1 (0.3%)

N.A. = Not applicable GP = General Practitioner * Question was only available if the patient stated to have experienced any health complaints during and or within 48 h from the last infusion

Fatigue was the most commonly reported health complaint (in 11 patients (9.5%), 4 classic infantile patients (23.5%), 1 childhood onset patient (6.6%) and 6 adult patients (7.3%)), and was most frequently reported during or within 24 h after infusion. In some patients, this complaint persisted until 24–48 h after infusion (in 3 classic infantile patients and 4 adult patients). Only one classic infantile patient reported fatigue solely after infusion, whereas all other patients who experienced fatigue after infusion also reported this during infusion. Headaches were the second most commonly reported health complaint, both during and after ERT, with it being reported in 9 patients (7.8%), 3 classic infantile patients (17.6%) and 6 adult patients (7.3%). Interestingly, headaches were more common the 24 h after infusion (reported 6 times in 2 classic infantile patients; 14.3% of complaints within the group, and 6 times in 6 adult patients; 16.2% of complaints within the group) than during infusion (2 times in 2 classic infantile patients; 6.5% of complaints within the group, 4 times in 4 adult patients; 11.8% of complaints within the group). As with fatigue, the prevalence of headaches decreased between the first 24 and 24–48 h post-infusion in both classic infantile patients (reported 3 times in 1 patient) and adult patients (reported 3 times in 3 patients). There were 1 classic infantile patient and two adult patients who experienced headaches only after but not during infusion.

Thirdly, feeling generally unwell was reported in 7 patients (6.0%), 4 classic infantile patients (23.5%) and 3 adult patients (3.7%). This was more common the 24 h after infusion in the infantile group (5 times in 3 classic infantile patients; 11.9% of complaints within the group) than during infusion (3 times in 3 patients; 9.7% of complaints within the group). In the adult group, feeling generally unwell decreased from 3 times in 3 patients during infusion (8.8% of complaints within the group) to 2 times in the same 2 patients 24 h after infusion (5.4% of complaints within the group). For both the classic infantile and the adult patients, the prevalence of feeling generally unwell went down 24–48 h after infusion.

Gastrointestinal complaints such as abdominal pain, diarrhoea, heartburn and vomiting were only reported after ERT; nausea was the only gastrointestinal complaint that was also reported during infusion.

For all 27 infusions in which patients reported health complaints, it was checked whether they had also been reported in the patient's medical file. Of these, health complaints were reported to the hospital in 4 instances (14.8%) in 2 classic infantile patients. These complaints were a skin rash, chills and a headache, fever, erythema and itching skin, and were assessed to be IARs. The remaining 85.2% of health complaints were not reported to the treating hospital by either the nurse or the patient.

Only in 4.9% of classic infantile (3 times in 3 patients), none of the atypical infantile, 4.5% of childhood-onset (2 times in 1 patient), and 2.6% of adult questionnaires (8 times in 7 patients), patients reported visiting the emergency GP, emergency room or being hospitalised in the week(s) after their last infusion (Table 2). None of these visits seemed to be related to the infusions; the itching reported by one patient was caused by scabies.

### Reporting of IARs and their management

The second part of our questionnaire consisted of questions about IARs occurring in the past three months (Additional files 1 and 2) to assess whether what patients experienced corresponds with what was reported to the hospital. On 96.2% of questionnaires, no IARs were reported by patients. In the classic infantile group, 3 patients indicated experiencing IARs on one or multiple of the four questionnaires. One patient reported having experienced multiple IARs on each questionnaire, 1 time 2 IARs, 1 time 3 IARs, 1 time 4 IARs and 1 time 6 IARs in the three months preceding each questionnaire. In this patient, 3 times 3 IARs and 1 time 4 IARs had been reported to the treating hospital. Another patient reported experiencing 3 IARs on one of the questionnaires; in this patient, 1 time 2 IARs had been reported to the hospital. The last patient indicated to have experienced 6 IARs over the previous three months on all four questionnaires, whereas 2 times 2 and 2 times 6 IARs had been reported to the hospital (Table 3).

**Table 3:** Infusion Associated Reactions

	Total	Classic infantile	Atypical infantile	Childhood-onset	Adult
Number of IARs past 3 months, n (% of questionnaires)					
1 infusion	1 (0.2%)	0 (0.0%)	0 (0.0%)	0 (0.0%)	1 (0.3%)
2 infusions	1 (0.2%)	1 (1.6%)	0 (0.0%)	0 (0.0%)	0 (0.0%)
3 infusions	2 (0.5%)	2 (3.3%)	0 (0.0%)	0 (0.0%)	0 (0.0%)
4 infusions	1 (0.2%)	1 (1.6%)	0 (0.0%)	0 (0.0%)	0 (0.0%)
6 infusions	9 (2.1%)	5 (8.2%)	0 (0.0%)	0 (0.0%)	4 (1.3%)
12 infusions	1 (0.2%)	0 (0.0%)	0 (0.0%)	0 (0.0%)	1 (0.3%)^#^
Never	407 (96.2%)	51 (83.6%)	8 (100.0%)	44 (100.0%)	304 (98.1%)
Missing	1 (0.2%)	1 (1.6%)	0 (0.0%)	0 (0.0%)	0 (0.0%)
Actions taken past 3 months in response to IAR, n (% of interventions)					
Infusion paused	7 (23.3%)	7 (28.0%)	N.A	N.A	0 (0.0%)
Infusion speed adapted	4 (13.3%)	4 (16.0%)	N.A	N.A	0 (0.0%)
Erasmus MC contacted	9 (30.0%)	7 (28.0%)	N.A	N.A	2 (40.0%)
Medication was given	9 (30.0%)	6 (24.0%)	N.A	N.A	3 (60.0%)
Admitted to hospital	1 (3.3%)	1 (4.0%)	N.A	N.A	0 (0.0%)
Did the IAR return after restarting the infusion^*^, n (% of paused infusions)					
Yes	0 (0.0%)	0 (0.0%)	N.A	N.A	N.A
No	7 (100.0%)	7 (100.0%)	N.A	N.A	N.A
Did the infusion reaction resolve due to the actions taken^**^, n (% of interventions)					
Yes	11 (78.6%)	8 (88.9%)	N.A	N.A	3 (60.0%)
No	1 (7.1%)	0 (0.0%)	N.A	N.A	1 (20.0%)
Don’t know	1 (7.1%)	0 (0.0%)	N.A	N.A	1 (20.0%)
Missing	1 (7.1%)	1 (11.1%)	N.A	N.A	0 (0.0%)
Actions taken to prevent IARs, n (% interventions)					
One of the infusion steps was adapted	3 (10.7%)	3 (15.0%)	N.A	N.A	0 (0.0%)
Infusion schedule completely adapted	7 (25.0%)	4 (20.0%)	N.A	N.A	3 (37.5%)
Premedication	10 (35.7%)	6 (30.0%)	N.A	N.A	4 (50.0%)
Next infusion in the hospital	7 (25.0%)	7 (35.0%)	N.A	N.A	0 (0.0%)
None	1 (3.6%)	0 (0.0%)	N.A	N.A	1 (12.5%)

N.A. = Not applicable * Question only available if the patients’ infusion had been paused in response to an IAR ** Question only available if actions were taken in response to IARs ^#^ Only 6 infusions were provided to this patient in the preceding three months

In the three classic infantile patients with IARs, 25 interventions in response to IARs were reported; multiple response options were possible because patients could have had multiple interventions to an infusion or multiple IARs that warranted different interventions. Pausing the infusion (7 times (28% of interventions)) and contacting the Erasmus MC (7 times (28% of interventions)) were the most common interventions, followed by giving medication (6 times (24% of interventions)) and adapting the infusion speed (4 times (16% of interventions)). One patient was admitted to the hospital (4% of interventions in the classic infantile group) after infusion due to fluid retention and defecation problems. None of the IARs warranted emergency care in the hospital. The interventions mentioned above resolved all IARs. To prevent IARs at subsequent infusions, the next infusion was given in the hospital (7 times (35.0% of interventions)), premedication was prescribed (6 times (30.0% of interventions)), the infusion schedule was either completely adapted (4 times (20.0% of interventions)), or only one step of the infusion schedule was adapted (3 times (15.0% of interventions)).

In the adult patient group, 3 patients indicated experiencing IARs on one or multiple questionnaires. One patient reported having experienced 12 IARs on one of the questionnaires (which could not be explained as only 6 infusions were provided in the preceding three months), and another patient reported having experienced 1 IAR on one questionnaire. The last patient reported having experienced 6 IARs on all 4 questionnaires. Only for this patient, 6 IARs had been reported to the hospital for all 4 three-month periods. This patient was known to have mild IARs, which were accepted in the home setting. No IARs had been reported to the treating hospital for the other patients. In total, 5 interventions in response to IARs were reported. Giving medication (3 times (60% of interventions)) and contacting the Erasmus MC (2 times (40% of interventions)) were the most common interventions. This resolved IARs in all but 1 case. One other patient reported not knowing whether it was the action taken that resolved the IAR. To prevent future IARs, most commonly, premedication was prescribed for the subsequent infusion (4 times (50% of interventions)), followed by either completely adapting the infusion schedule (3 times (37.5% of interventions)) or adapting one of the infusion steps (1 (12.5% of interventions)).

No IARs were reported during the one-year follow-up in the atypical infantile and childhood-onset groups.

## Discussion

The results of this prospective study demonstrate that home-based ERT in Pompe disease can be safely implemented in both classic infantile Pompe patients receiving high dosage ERT (40 mg/kg/week) and late-onset patients as few, mostly mild, symptoms were reported during or after infusion. Only a few IARs occurred during the study period, and none needed emergency clinical care. This is in line with a previous long-term follow-up study, which demonstrated that alglucosidase alfa can be safely administered at home in adult patients with Pompe disease [23]. Despite home-infusions not being included in the summary of product characteristics (SmPC) due to the risk of anaphylaxis, severe allergic and immune-mediated reactions, and risk of cardiorespiratory failure in response to alglucosidase alfa [21, 22], home-infusion with alglucosidase alfa should be considered. Patients’ quality of life is negatively impacted by time-consuming infusions [27]; home-based ERT can diminish the burden of treatment, increase patient flexibility and autonomy [20], and is, thus, a more patient-centred approach to ERT.

Most patients in this study indicated that they did not experience any health complaints before infusion. The reported health complaints were mostly mild. Fatigue, headaches and feeling generally unwell were most frequently reported during and within 24 h after infusion; symptoms subdued after that in most patients. Whether the fatigue or headaches were caused by the alglucosidase alfa infusion (i.e. IARs), Pompe disease itself or environmental factors (such as having a nurse by your side for several hours or being limited in your activities) remains unknown. Fatigue is a common symptom of Pompe disease and has been reported in 24% up to 85% of adult Pompe patients [28–31]. Hence, unsurprisingly, fatigue was reported in 9.5% of patients during or after infusion in our study. Possible explanations for fatigue in Pompe disease include peripheral fatigue from both skeletal and respiratory muscle weakness [29], with respiratory muscle weakness potentially leading to daytime sleepiness and fatigue [29, 32, 33]. Central fatigue may also play a role, with a sense of cognitive, psychosocial or mental exhaustion leading to fatigue [28, 29]. We postulate that fatigue is multifactorial and that both peripheral fatigue as well as central fatigue may contribute to fatigue in Pompe disease [29, 34]. It has been reported that fatigue improves significantly during ERT in several patient subgroups [28]. To manage fatigue and treat Pompe disease in general, treatment options such as rehabilitation, exercise and nutritional interventions should be considered [28, 35], as these can be complementary to ERT [35–37]. In addition to fatigue, headaches were reported relatively often during or after infusion (in 7.6% of patients). However, this may not be due to ERT per se. It should be noted that some Pompe patients experience headaches caused by nocturnal hypoventilation due to diaphragmatic weakness, though morning headaches upon waking up are more exemplary of this [33]. A prior study in a cohort of 73 patients demonstrated that 15% of patients with IARs experienced fatigue, and 23% of patients with IARs experienced headaches [24]. However, fatigue and headaches constituted a smaller fraction of IARs in this study [24] compared with the higher fraction of these health complaints in our study, possibly due to our study method; using a home infusion cohort and the primary outcome measure being symptoms reported by patients rather than IARs reported to the hospital.

Interestingly, gastrointestinal complaints such as abdominal pain, diarrhoea, heartburn and vomiting were only reported within 24–48 h after infusion but not during infusion. We could not find an explanation for the mere occurrence of gastrointestinal complaints after the administration of ERT. Still, temporary worsening of GI symptoms has been previously described within 48 h of ERT infusion [38]. We cannot rule out that these are side effects of ERT [39]. However, gastrointestinal complaints have also been reported to be part of the symptom complex in Pompe disease [40, 41], for glycogen accumulates in smooth muscle throughout the gastrointestinal tract [40, 42–49]. Overall, ERT has been reported to lead to long-term improvements in GI symptoms [40, 41]. All symptoms that patients in this study reported are also included in the summary of product characteristics (SMPC) of alglucosidase alfa [22].

Of the health complaints reported in this study, only four (14.8%) were reported to the treating hospital as an IAR. Thus, there was underreporting of health complaints to the Erasmus MC University Medical Center. However, these health complaints were all either mild, pre-existing, had an unclear relation to ERT, or occurred after infusion. Though mild symptoms, such as fatigue or headache, do not pose an immediate health risk to patients, these complaints may be important to the patient. This highlights the importance of patient-reported outcomes in the evaluation of medical care, as these may help clinicians address reported symptoms better and generate data that are truly relevant to the patient and complementary to clinical data [31]. To improve individual patient care, it is thus essential to inquire whether the patient experiences minor health complaints around infusions during outpatient clinic visits. If any complaints are reported, then the burden of these complaints should be explored, and medical action should be taken if necessary.

Interestingly, of the patients who indicated feeling sick or ill before infusion, 38.5% experienced health complaints during infusion, versus 5.3% of patients who were not sick or ill before infusion. This may imply that patients who are sick or ill prior to administration of ERT may be at an increased risk to develop health complaints during infusion, possibly due to activation of the immune system. However, more research is needed in this regard.

Patients reported slightly more IARs on average than were reported to the treating hospital. However, some patients reported fewer IARs in the past three months than had been reported to the treating hospital. This difference could be explained by recall bias, as patients had to answer retrospectively how many IARs they had experienced in the past three months. All IARs could be managed at home and did not require immediate hospital evaluation. In the Netherlands, we have learned from experience that pausing the infusion causes symptoms to disappear in the majority of IARs. As such, this is the first step in the current SOP for IAR management. If needed, the next step is to administer antipyretics, antihistamines and/or corticosteroids, depending on the type of IAR. This is consistent with what has been described in literature on effective management of IARs in enzyme replacement therapies [24, 50–53]. Interestingly, in the adult group, none of the patients reported that the infusion was paused due to IARs, which is the standard first response in our home infusion SOP. This could be explained by the fact that these IARs were very mild and were accepted as such (i.e. one adult patient with repetitive IARs experienced transient skin rashes at the highest infusion rate). Although most patients did not experience IARs, five out of 116 patients (3 classic infantile, and 2 adults; 17.6% and 2.4% of patient within these groups respectively) experienced multiple IARs. The higher prevalence of both health complaints, IARs and interventions in response to IARs within the classic infantile group could be explained by the fact that classic infantile patients have less than 1% residual activity of alpha-glucosidase [1], making them more likely to have an adverse response to ERT [54]. Another explanatory factor may be the higher dosage and frequency of ERT given to classic infantile patients (40 mg/kg/week versus 20 mg/kg/biweekly). However, a previous study reported that patients treated with a high dosage did not seem to have more IARs than those treated with the standard recommended dosage [55]. Additionally, the four patients in the childhood-onset group receiving a dosage higher than the standard recommended dosage of ERT did not report any IARs. Further investigation on the role of ERT dosage in the aetiology of IARs is needed [56].

Although our study had a high response rate of 88.1%, there was a relatively large difference in response rates between groups, with the childhood-onset patients having a substantially lower response rate of 68.8% than the other groups. This is likely explained by the fact that most patients in this group are adolescents or young adults, typically with a higher rate of non-response [57], limiting the interpretation of data in this group.

## Conclusions

This study demonstrates that home-based ERT in Pompe disease is safe across the whole phenotypic spectrum. We did not encounter any new ERT-related health problems. Insights gained from this study can be used as a base for the implementation of home infusions in other countries and for further optimisation of patient care with home-based ERT in Pompe disease. Mild symptoms that occur during or after infusion may still be relevant to the patient, even if they do not pose a health risk.

## Supplementary Information

**Additional file 1**. Flow chart of the questionnaire.

**Additional file 2.** This file contains the questionnaires used to conduct this study. It contains three questionnaires. One for children < 12 years of age, one for children ≥12 years of age, and one for adults ≥18 years of age.

**Additional file 3**. Symptoms occurring during or up to 48 hours after infusion.

## Data Availability

The dataset supporting the conclusions of this article is available upon request to the corresponding author from any qualified investigator for the sole purpose of replicating procedures and results presented in the Article, in agreement with EU legislation on the general data protection regulation.

## Abbreviations

GAA: Acid α-glucosidase
EC number: Enzyme commission number
ERT: Enzyme replacement therapy
LVMI: Left ventricular mass index
IARs: Infusion associated reactions
SOP: Standard operating procedure
WMO: Wet medisch-wetenschappelijk onderzoek met mensen (Medical research involving human subjects act)
N.A.: Not applicable
SmPC: Summary of product characteristics
i.e.: Id est (that is)

## References

[1] van der Ploeg AT, Reuser AJ (2008). Pompe's disease. Lancet.

[2] Güngör D, Reuser AJ (2013). How to describe the clinical spectrum in Pompe disease?. Am J Med Genet A.

[3] Slonim AE, Bulone L, Ritz S, Goldberg T, Chen A, Martiniuk F (2000). Identification of two subtypes of infantile acid maltase deficiency. J Pediatr.

[4] Winkel LP, Hagemans ML, van Doorn PA, Loonen MC, Hop WJ, Reuser AJ (2005). The natural course of non-classic Pompe's disease; a review of 225 published cases. J Neurol.

[5] In 't Groen SLM, de Faria DOS, Iuliano A, van den Hout JMP, Douben H, Dijkhuizen T, et al. Novel GAA Variants and Mosaicism in Pompe Disease Identified by Extended Analyses of Patients with an Incomplete DNA Diagnosis. Mol Ther Methods Clin Dev. 2020;17:337–48. 10.1016/j.omtm.2019.12.016 PMC7013133 32071926

[6] Van den Hout H, Reuser AJ, Vulto AG, Loonen MC, Cromme-Dijkhuis A, Van der Ploeg AT (2000). Recombinant human alpha-glucosidase from rabbit milk in Pompe patients. Lancet.

[7] Kishnani PS, Corzo D, Nicolino M, Byrne B, Mandel H, Hwu WL (2007). Recombinant human acid [alpha]-glucosidase: major clinical benefits in infantile-onset Pompe disease. Neurology.

[8] Kishnani PS, Corzo D, Leslie ND, Gruskin D, Van der Ploeg A, Clancy JP (2009). Early treatment with alglucosidase alpha prolongs long-term survival of infants with Pompe disease. Pediatr Res.

[9] van Capelle CI, Poelman E, Frohn-Mulder IM, Koopman LP, van den Hout JMP, Regal L (2018). Cardiac outcome in classic infantile Pompe disease after 13years of treatment with recombinant human acid alpha-glucosidase. Int J Cardiol.

[10] Ditters IAM, Huidekoper HH, Kruijshaar ME, Rizopoulos D, Hahn A, Mongini TE (2022). Effect of alglucosidase alfa dosage on survival and walking ability in patients with classic infantile Pompe disease: a multicentre observational cohort study from the European Pompe Consortium. Lancet Child Adolesc Health.

[11] van der Ploeg AT, Clemens PR, Corzo D, Escolar DM, Florence J, Groeneveld GJ (2010). A randomized study of alglucosidase alfa in late-onset Pompe's disease. N Engl J Med.

[12] Strothotte S, Strigl-Pill N, Grunert B, Kornblum C, Eger K, Wessig C (2010). Enzyme replacement therapy with alglucosidase alfa in 44 patients with late-onset glycogen storage disease type 2: 12-month results of an observational clinical trial. J Neurol.

[13] Angelini C, Semplicini C, Ravaglia S, Bembi B, Servidei S, Pegoraro E (2012). Observational clinical study in juvenile-adult glycogenosis type 2 patients undergoing enzyme replacement therapy for up to 4 years. J Neurol.

[14] van der Ploeg AT, Barohn R, Carlson L, Charrow J, Clemens PR, Hopkin RJ (2012). Open-label extension study following the Late-Onset Treatment Study (LOTS) of alglucosidase alfa. Mol Genet Metab.

[15] Gungor D, Kruijshaar ME, Plug I, D'Agostino RB, Hagemans ML, van Doorn PA (2013). Impact of enzyme replacement therapy on survival in adults with Pompe disease: results from a prospective international observational study. Orphanet J Rare Dis.

[16] Anderson LJ, Henley W, Wyatt KM, Nikolaou V, Waldek S, Hughes DA (2014). Effectiveness of enzyme replacement therapy in adults with late-onset Pompe disease: results from the NCS-LSD cohort study. J Inherit Metab Dis.

[17] Schoser B, Stewart A, Kanters S, Hamed A, Jansen J, Chan K (2017). Survival and long-term outcomes in late-onset Pompe disease following alglucosidase alfa treatment: a systematic review and meta-analysis. J Neurol.

[18] van der Meijden JC, Kruijshaar ME, Harlaar L, Rizopoulos D, van der Beek N, van der Ploeg AT. Long-term follow-up of 17 patients with childhood Pompe disease treated with enzyme replacement therapy. J Inherit Metab Dis. 2018. 10.1007/s10545-018-0166-3 PMC6326992 29556838

[19] Harlaar L, Hogrel J-Y, Perniconi B, Kruijshaar ME, Rizopoulos D, Taouagh N (2019). Large variation in effects during 10 years of enzyme therapy in adults with Pompe disease. Neurology.

[20] Perraudin C, Bourdin A, Vicino A, Kuntzer T, Bugnon O, Berger J (2020). Home-based subcutaneous immunoglobulin for chronic inflammatory demyelinating polyneuropathy patients: a Swiss cost-minimization analysis. PLoS ONE.

[21] FDA. Highlights of prescribing information MYOZYME® (alglucosidase alfa) injectable for intravenous infusion: FDA [updated 05–2019. Available from: https://www.accessdata.fda.gov/drugsatfda_docs/label/2014/125141s219lbl.pdf.

[22] Myozyme: EPAR - Product information: European Medicines Agency; 2022 [updated 25–08–2022. Available from: https://www.ema.europa.eu/en/documents/product-information/myozyme-epar-product-information_en.pdf.

[23] Ditters I, van Kooten H, van der Beek N, van der Ploeg A, van den Hout H, Huidekoper H (2022). eP030502 Safety of home-based infusion of alglucosidase alfa in adults with late-onset Pompe disease. J Neuromuscul Dis..

[24] de Vries JM, Kuperus E, Hoogeveen-Westerveld M, Kroos MA, Wens SCA, Stok M (2017). Pompe disease in adulthood: effects of antibody formation on enzyme replacement therapy. Genet Med.

[25] van der Ploeg AT, Kruijshaar ME, Toscano A, Laforet P, Angelini C, Lachmann RH (2017). European consensus for starting and stopping enzyme replacement therapy in adult patients with Pompe disease: a 10-year experience. Eur J Neurol.

[26] MC E. Gemstracker, (GEneric Medical Survey Tracker): Erasmus MC, Rotterdam; 2022. Available from: https://gemstracker.org/.

[27] Güngör D, Kruijshaar ME, Plug I, Rizopoulos D, Kanters TA, Wens SC (2016). Quality of life and participation in daily life of adults with Pompe disease receiving enzyme replacement therapy: 10 years of international follow-up. J Inherit Metab Dis.

[28] Güngör D, de Vries JM, Brusse E, Kruijshaar ME, Hop WC, Murawska M (2013). Enzyme replacement therapy and fatigue in adults with Pompe disease. Mol Genet Metab.

[29] Hagemans ML, van Schie SP, Janssens AC, van Doorn PA, Reuser AJ, van der Ploeg AT (2007). Fatigue: an important feature of late-onset Pompe disease. J Neurol.

[30] Hagemans ML, Winkel LP, Van Doorn PA, Hop WJ, Loonen MC, Reuser AJ (2005). Clinical manifestation and natural course of late-onset Pompe's disease in 54 Dutch patients. Brain.

[31] van der Meijden JC, Güngör D, Kruijshaar ME, Muir AD, Broekgaarden HA, van der Ploeg AT (2015). Ten years of the international Pompe survey: patient reported outcomes as a reliable tool for studying treated and untreated children and adults with non-classic Pompe disease. J Inherit Metab Dis.

[32] Shah NM, Sharma L, Ganeshamoorthy S, Kaltsakas G (2020). Respiratory failure and sleep-disordered breathing in late-onset Pompe disease: a narrative review. J Thorac Dis.

[33] Boentert M, Karabul N, Wenninger S, Stubbe-Dräger B, Mengel E, Schoser B (2015). Sleep-related symptoms and sleep-disordered breathing in adult Pompe disease. Eur J Neurol.

[34] Chaudhuri A, Behan PO (2004). Fatigue in neurological disorders. Lancet.

[35] Favejee MM, van den Berg LE, Kruijshaar ME, Wens SC, Praet SF, Pim Pijnappel WW (2015). Exercise training in adults with Pompe disease: the effects on pain, fatigue, and functioning. Arch Phys Med Rehabil.

[36] Angelini C. Exercise, nutrition and enzyme replacement therapy are efficacious in adult Pompe patients: report from EPOC Consortium. Eur J Transl Myol. 2021;31(2). 10.4081/ejtm.2021.9798 PMC8274227 33942602

[37] van den Berg LE, Favejee MM, Wens SC, Kruijshaar ME, Praet SF, Reuser AJ (2015). Safety and efficacy of exercise training in adults with Pompe disease: evalution of endurance, muscle strength and core stability before and after a 12 week training program. Orphanet J Rare Dis.

[38] Korlimarla A, Lim JA, McIntosh P, Zimmerman K, Sun BD, Kishnani PS. New insights into gastrointestinal involvement in late-onset pompe disease: lessons learned from bench and bedside. J Clin Med. 2021;10(15). 10.3390/jcm10153395 PMC8347662 34362174

[39] Karabul N, Skudlarek A, Berndt J, Kornblum C, Kley RA, Wenninger S (2014). Urge incontinence and gastrointestinal symptoms in adult patients with pompe disease: a cross-sectional survey. JIMD Rep.

[40] Bernstein DL, Bialer MG, Mehta L, Desnick RJ (2010). Pompe disease: dramatic improvement in gastrointestinal function following enzyme replacement therapy. A report of three later-onset patients. Mol Genet Metab.

[41] Pardo J, García-Sobrino T, López-Ferreiro A (2015). Gastrointestinal symptoms in late-onset Pompe disease: early response to enzyme replacement therapy. J Neurol Sci.

[42] Hobson-Webb LD, Proia AD, Thurberg BL, Banugaria S, Prater SN, Kishnani PS (2012). Autopsy findings in late-onset Pompe disease: a case report and systematic review of the literature. Mol Genet Metab.

[43] van der Walt JD, Swash M, Leake J, Cox EL (1987). The pattern of involvement of adult-onset acid maltase deficiency at autopsy. Muscle Nerve.

[44] Bijvoet AG, Van Hirtum H, Kroos MA, Van de Kamp EH, Schoneveld O, Visser P (1999). Human acid alpha-glucosidase from rabbit milk has therapeutic effect in mice with glycogen storage disease type II. Hum Mol Genet.

[45] Bijvoet AG, Van Hirtum H, Vermey M, Van Leenen D, Van Der Ploeg AT, Mooi WJ (1999). Pathological features of glycogen storage disease type II highlighted in the knockout mouse model. J Pathol.

[46] Thurberg BL, Lynch Maloney C, Vaccaro C, Afonso K, Tsai AC, Bossen E (2006). Characterization of pre- and post-treatment pathology after enzyme replacement therapy for Pompe disease. Lab Invest.

[47] Pena LD, Proia AD, Kishnani PS (2015). Postmortem findings and clinical correlates in individuals with infantile-onset Pompe disease. JIMD Rep.

[48] Chan J, Desai AK, Kazi ZB, Corey K, Austin S, Hobson-Webb LD (2017). The emerging phenotype of late-onset Pompe disease: a systematic literature review. Mol Genet Metab.

[49] McCall AL, Salemi J, Bhanap P, Strickland LM, Elmallah MK (2018). The impact of Pompe disease on smooth muscle: a review. J Smooth Muscle Res.

[50] Burrow TA, Hopkin RJ, Leslie ND, Tinkle BT, Grabowski GA (2007). Enzyme reconstitution/replacement therapy for lysosomal storage diseases. Curr Opin Pediatr.

[51] Regnery C, Kornblum C, Hanisch F, Vielhaber S, Strigl-Pill N, Grunert B (2012). 36 months observational clinical study of 38 adult Pompe disease patients under alglucosidase alfa enzyme replacement therapy. J Inherit Metab Dis.

[52] Capanoglu M, Dibek Misirlioglu E, Azkur D, Vezir E, Guvenir H, Gunduz M (2016). IgE-mediated hypersensitivity and desensitisation with recombinant enzymes in Pompe disease and type I and type VI mucopolysaccharidosis. Int Arch Allergy Immunol.

[53] El-Gharbawy AH, Mackey J, DeArmey S, Westby G, Grinnell SG, Malovrh P (2011). An individually, modified approach to desensitize infants and young children with Pompe disease, and significant reactions to alglucosidase alfa infusions. Mol Genet Metab.

[54] Kishnani PS, Goldenberg PC, DeArmey SL, Heller J, Benjamin D, Young S (2010). Cross-reactive immunologic material status affects treatment outcomes in Pompe disease infants. Mol Genet Metab.

[55] van Gelder CM, Poelman E, Plug I, Hoogeveen-Westerveld M, van der Beek N, Reuser AJJ (2016). Effects of a higher dose of alglucosidase alfa on ventilator-free survival and motor outcome in classic infantile Pompe disease: an open-label single-center study. J Inherit Metab Dis.

[56] Poelman E, van den Dorpel J, Hoogeveen-Westerveld M, van den Hout J, van der Giessen LJ, van der Beek N, et al. Effects of higher and more frequent dosing of alglucosidase alfa and immunomodulation on long-term clinical outcome of classic infantile Pompe patients. J Inherit Metab Dis. 2020. 10.1002/jimd.12268 PMC7689828 32506446

[57] Cheung KL, Ten Klooster PM, Smit C, de Vries H, Pieterse ME (2017). The impact of non-response bias due to sampling in public health studies: A comparison of voluntary versus mandatory recruitment in a Dutch national survey on adolescent health. BMC Public Health.

