# Supplementary material for: Home-based enzyme replacement therapy in children and adults with Pompe disease; a prospective study
Source: Orphanet J Rare Dis. 2023 May 8;18:108. doi: 10.1186/s13023-023-02715-4 (PMC10169363; doi:10.1186/s13023-023-02715-4)
Supplement: Supplementary file 1 — Additional file 1. Flow chart of the questionnaire. [file 13023_2023_2715_MOESM1_ESM.pptx]

## Slide 1
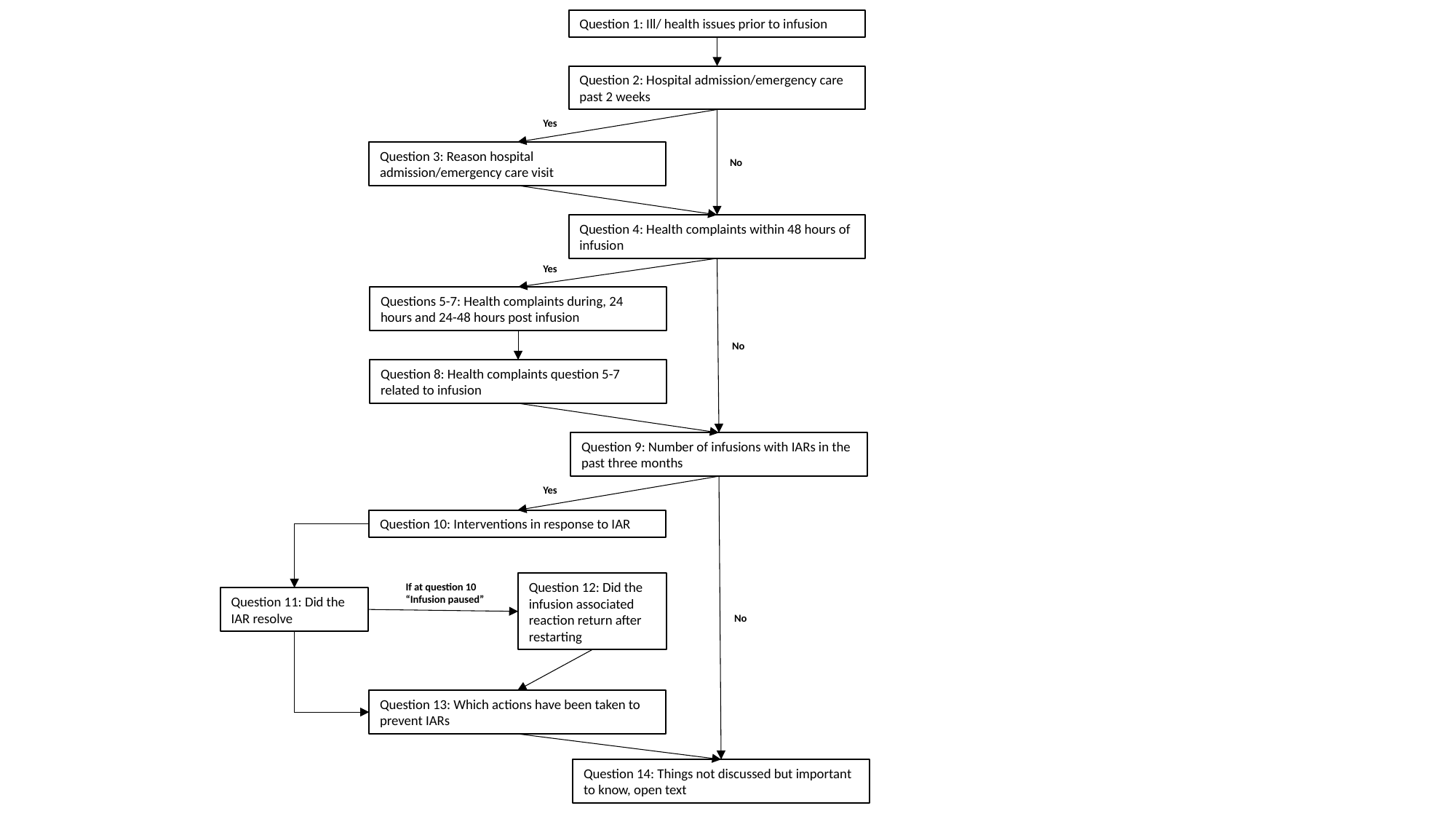

Question 1: Ill/ health issues prior to infusion
Question 2: Hospital admission/emergency care past 2 weeks
Yes
Question 3: Reason hospital admission/emergency care visit
No
Question 4: Health complaints within 48 hours of infusion
Yes
Questions 5-7: Health complaints during, 24 hours and 24-48 hours post infusion
No
Question 8: Health complaints question 5-7 related to infusion
Question 9: Number of infusions with IARs in the past three months
Yes
Question 10: Interventions in response to IAR
Question 12: Did the infusion associated reaction return after restarting
If at question 10
“Infusion paused”
Question 11: Did the IAR resolve
No
Question 13: Which actions have been taken to prevent IARs
Question 14: Things not discussed but important to know, open text
07/05/2023
1
