# Supplementary material for: Home-based enzyme replacement therapy in children and adults with Pompe disease; a prospective study
Source: Orphanet J Rare Dis. 2023 May 8;18:108. doi: 10.1186/s13023-023-02715-4 (PMC10169363; doi:10.1186/s13023-023-02715-4)
Supplement: Supplementary file 2 — Additional file 2. This file contains the questionnaires used to conduct this study. It contains three questionnaires. One for children < 12 years of age, one for children ≥12 years of age, and one for adults ≥18 years of age. [file 13023_2023_2715_MOESM2_ESM.docx]

**Additional file 2: Questionnaires**

***Questionnaire children <12 years of age.***

**Prospective questionnaire parents of children home infusion project**

General introduction

Thank you for your participation in Erasmus MC's home infusion project. The aim of this project is to gain a better understanding of the patient population receiving home infusions for Pompe disease, whether patients have infusion reactions and how these are managed.

This short questionnaire will ask a number of questions about your child's infusion therapy, including what symptoms he/she experienced in the period surrounding the infusion. You can involve your child in completing the questionnaire. From the age of 12, the questionnaire can be completed entirely together with your child.

We thank you in advance for your cooperation; it is greatly appreciated.

1. ***Has your child been ill/has your child experienced any health complaints prior to administration of the last infusion that might be relevant for safe administration?***

□ Yes, please specify:

______________________________________________________________________________________________________________________________________________________

□ No

□ Don’t know

1. ***Has your child been hospitalized since his/her last infusion/has your child visited A&E / the emergency GP?***

□ Yes

□ No

If “Yes” has been answered to question 2, question 3 appears.

1. ***For what reason has your child been hospitalized / did your child visit A&E / the emergency GP?***

*______________________________________________________________________________________________________________________________________________________*

1. ***Did your child experience any health complaints during and/or within 48 hours of their last infusion?***

□ Yes

□ No

If “Yes” has been answered to question 4, questions 5 through 8 will appear.

By means of the following three questions, we would like to find out what symptoms your child experienced during his/her last infusion (question 5), the first 24 hours after his/her last infusion (question 6) and 24-48 hours after his/her last infusion (question 7).

1. ***Which of the following symptoms did your child experience during his/her last infusion? (multiple answers allowed)***

Please tick all options that apply to your child

□ None

□ Generally unwell

□ Elevated body temperature (37.5-38.5 C)

□ Fever (temperature >38.5 C)

□ Chills

□ Excessive sweating

□ Pallor

□ Blushing

□ Itching (Please tick all that apply):

| □ Neck | □ Shoulders | □ Back |
| --- | --- | --- |
| □ Chest | □ Upper arms | □ Lower arms |
| □ Abdomen | □ Hands | □ Upper legs |
| □ Lower legs | □ Feet | □ Face |

□ Swollen lip/tongue

□ Skin rash/ hives/ nettle rash (Please tick all that apply):

| □ Neck | □ Shoulders | □ Back |
| --- | --- | --- |
| □ Chest | □ Upper arms | □ Lower arms |
| □ Abdomen | □ Hands | □ Upper legs |
| □ Lower legs | □ Feet | □ Face |

□ Reaction at the infusion site (such as pain, swelling, hardening)

□ Red eyes

□ Oedema (swelling)

| □ Neck | □ Throat | □ Back |
| --- | --- | --- |
| □ Chest | □ Upper arms | □ Lower arms |
| □ Abdomen | □ Hands | □ Upper legs |
| □ Lower legs | □ Feet | □ Face |

□ Nausea

□ Vomiting

□ Diarrhoea

□ Abdominal pain

□ Stomach pain/ heartburn

□ Coughing

□ Rapid breathing

□ Wheezing

□ Shortness of breath

□ Rapid heart rate

□ Slow heart rate

□ Palpitations

□ Chest pain

□ Irritability

□ Restlessness

□ Dizziness

□ Headache

□ Fatigue

□ Trembling

□ Stinging/ tingling/ numb feeling in one or more limbs

□ Stinging feeling in the mouth/the face

□ Joint pain

□ Muscle spasms/muscle cramps (Please tick all that apply):

| □ Neck | □ Shoulders | □ Back |
| --- | --- | --- |
| □ Chest | □ Upper arms | □ Lower arms |
| □ Abdomen | □ Hands | □ Upper legs |
| □ Lower legs | □ Feet |  |

□ Muscle pain (Please tick all that apply):

| □ Neck | □ Shoulders | □ Back |
| --- | --- | --- |
| □ Chest | □ Upper arms | □ Lower arms |
| □ Abdomen | □ Hands | □ Upper legs |
| □ Lower legs | □ Feet |  |

□ Don’t know

□ Other, please specify: ________________________________________________________________________________________________________________________________________________

1. ***Which of the following symptoms did your child experience within 24 hours of his/her last infusion? (multiple answers allowed)***

Please tick all options that apply to your child

□ None

□ Generally unwell

□ Elevated body temperature (37.5-38.5 C)

□ Fever (temperature >38.5 C)

□ Chills

□ Excessive sweating

□ Pallor

□ Blushing

□ Itching (Please tick all that apply):

| □ Neck | □ Shoulders | □ Back |
| --- | --- | --- |
| □ Chest | □ Upper arms | □ Lower arms |
| □ Abdomen | □ Hands | □ Upper legs |
| □ Lower legs | □ Feet | □ Face |

□ Swollen lip/tongue

□ Skin rash/ hives/ nettle rash (Please tick all that apply):

| □ Neck | □ Shoulders | □ Back |
| --- | --- | --- |
| □ Chest | □ Upper arms | □ Lower arms |
| □ Abdomen | □ Hands | □ Upper legs |
| □ Lower legs | □ Feet | □ Face |

□ Reaction at the infusion site (such as pain, swelling, hardening)

□ Red eyes

□ Oedema (swelling)

| □ Neck | □ Throat | □ Back |
| --- | --- | --- |
| □ Chest | □ Upper arms | □ Lower arms |
| □ Abdomen | □ Hands | □ Upper legs |
| □ Lower legs | □ Feet | □ Face |

□ Nausea

□ Vomiting

□ Diarrhoea

□ Abdominal pain

□ Stomach pain/ heartburn

□ Coughing

□ Rapid breathing

□ Wheezing

□ Shortness of breath

□ Rapid heart rate

□ Slow heart rate

□ Palpitations

□ Chest pain

□ Irritability

□ Restlessness

□ Dizziness

□ Headache

□ Fatigue

□ Trembling

□ Stinging/ tingling/ numb feeling in one or more limbs

□ Stinging feeling in the mouth/the face

□ Joint pain

□ Muscle spasms/ muscle cramps (Please tick all that apply):

| □ Neck | □ Shoulders | □ Back |
| --- | --- | --- |
| □ Chest | □ Upper arms | □ Lower arms |
| □ Abdomen | □ Hands | □ Upper legs |
| □ Lower legs | □ Feet |  |

□ Muscle pain (Please tick all that apply):

| □ Neck | □ Shoulders | □ Back |
| --- | --- | --- |
| □ Chest | □ Upper arms | □ Lower arms |
| □ Abdomen | □ Hands | □ Upper legs |
| □ Lower legs | □ Feet |  |

□ Don’t know

□ Other, please specify: ________________________________________________________________________________________________________________________________________________

1. ***Which of the following symptoms did your child experience within 24-48 hours of his/her last infusion? (multiple answers allowed)***

Please tick all options that apply to your child

□ None

□ Generally unwell

□ Elevated body temperature (37.5-38.5 C)

□ Fever (temperature >38.5 C)

□ Chills

□ Excessive sweating

□ Pallor

□ Blushing

□ Itching (Please tick all that apply):

| □ Neck | □ Shoulders | □ Back |
| --- | --- | --- |
| □ Chest | □ Upper arms | □ Lower arms |
| □ Abdomen | □ Hands | □ Upper legs |
| □ Lower legs | □ Feet | □ Face |

□ Swollen lip/tongue

□ Skin rash/ hives/ nettle rash (Please tick all that apply):

| □ Neck | □ Shoulders | □ Back |
| --- | --- | --- |
| □ Chest | □ Upper arms | □ Lower arms |
| □ Abdomen | □ Hands | □ Upper legs |
| □ Lower legs | □ Feet | □ Face |

□ Reaction at the infusion site (such as pain, swelling, hardening)

□ Red eyes

□ Oedema (swelling)

| □ Neck | □ Throat | □ Back |
| --- | --- | --- |
| □ Chest | □ Upper arms | □ Lower arms |
| □ Abdomen | □ Hands | □ Upper legs |
| □ Lower legs | □ Feet | □ Face |

□ Nausea

□ Vomiting

□ Diarrhoea

□ Abdominal pain

□ Stomach pain/ heartburn

□ Coughing

□ Rapid breathing

□ Wheezing

□ Shortness of breath

□ Rapid heart rate

□ Slow heart rate

□ Palpitations

□ Chest pain

□ Irritability

□ Restlessness

□ Dizziness

□ Headache

□ Fatigue

□ Trembling

□ Stinging/ tingling/ numb feeling in one or more limbs

□ Stinging feeling in the mouth/the face

□ Joint pain

□ Muscle spasms/ muscle cramps (Please tick all that apply):

| □ Neck | □ Shoulders | □ Back |
| --- | --- | --- |
| □ Chest | □ Upper arms | □ Lower arms |
| □ Abdomen | □ Hands | □ Upper legs |
| □ Lower legs | □ Feet |  |

□ Muscle pain (Please tick all that apply):

| □ Neck | □ Shoulders | □ Back |
| --- | --- | --- |
| □ Chest | □ Upper arms | □ Lower arms |
| □ Abdomen | □ Hands | □ Upper legs |
| □ Lower legs | □ Feet |  |

□ Don’t know

□ Other, please specify: ________________________________________________________________________________________________________________________________________________

1. ***Do you think the symptoms listed in questions 5 through 7 have anything to do with the infusion?***

□ Yes

□ No

□ Maybe

1. ***In the past 3 months, my child has had an infusion reaction during the following number of infusions:***

□ Never

□ 1 infusion

□ 2 infusions

□ 3 infusions

□ 4 infusions

□ 5 or more infusions

□ Every infusion

If the patient has had at least 1 infusion reaction in the past three months, questions 10 to 13 will appear.

1. ***What actions were taken in the last 3 months as a result of an infusion reaction? (multiple response options possible)***

□ Infusion stopped completely

□ Infusion paused

□ The infusion speed was adapted

□ A local hospital/ the GP was contacted

□ The Erasmus MC was contacted

□ An acute intervention was needed

□ My child was admitted to hospital

□ Medication was given, if yes, what medication? ________________________________________________________________________________________________________________________________________________

1. ***Did the infusion reaction go away as a result of the intervention mentioned in question 11?***

□ Yes

□ No

□ Don’t know

If the answer option "Infusion paused" is selected at question 10, then question 12 will appear.

1. ***Did the infusion reaction return after restarting the infusion? (if your child had multiple infusion reactions, fill in “yes” if the reaction returned at least once after restarting the infusion)***

□ Yes

□ No

□ Don’t know

1. ***What actions were taken after the occurrence of the infusion reaction(s) to prevent its recurrence in subsequent infusions? (multiple answers allowed)***

□ One of the infusion steps was adapted

□ The entire infusion schedule was adapted

□ My child receives medication prior to infusion

□ My child has discontinued therapy

□ The subsequent infusion was given in the hospital

1. ***Are there any topics that were not discussed in this questionnaire but that you feel are important for us to know?***

______________________________________________________________________________________________________________________________________________________

***Questionnaire children ≥12 years of age.***

**Prospective questionnaire children 12 to 18 years home infusion project**

General introduction

Thank you very much for participating in Erasmus MC's study on home infusion in Pompe disease. This study aims to gain a better understanding of enzyme therapy in the home situation. We will also investigate whether patients have infusion reactions (a reaction of the body to the protein (enzyme) you receive through the infusion) and how these are handled.

In this short questionnaire, we will ask you some questions about infusion therapy for Pompe disease. The questions are about symptoms you may have experienced during/before/after the infusion.

We thank you in advance for taking part in the survey, we appreciate your participation.

1. ***Have you been ill/have you experienced health issues before receiving the infusion that could cause the safety of the infusion to be reduced?***

□ Yes, please specify:

______________________________________________________________________________________________________________________________________________________

□ No

□ Don’t know

1. ***Have you been hospitalized since your last infusion/ have you been to A&E / the emergency GP?***

□ Yes

□ No

If “Yes” has been answered to question 2, question 3 appears.

1. ***For what reason have you been you hospitalized/ why did you visit A&E / the emergency GP?***

*_____________________________________________________________________________________________________________________________________________________****_***

1. ***Did you experience any health complaints during and/or within 48 hours of your last infusion?***

□ Yes

□ No

If “Yes” has been answered to question 4, questions 5 through 8 will appear.

By means of the following three questions, we would like find out what symptoms you experienced during your last infusion (question 5), the first 24 hours after your last infusion (question 6) and 24-48 hours after your last infusion (question 7).

1. ***Which of the following symptoms did you experience during your last infusion?***

***(multiple answers allowed)***

Please tick all options that apply to you

□ None

□ Generally unwell

□ Elevated body temperature (37.5-38.5 C)

□ Fever (temperature >38.5 C)

□ Chills

□ Excessive sweating

□ Pallor

□ Blushing

□ Itching (Please tick all that apply):

| □ Neck | □ Shoulders | □ Back |
| --- | --- | --- |
| □ Chest | □ Upper arms | □ Lower arms |
| □ Abdomen | □ Hands | □ Upper legs |
| □ Lower legs | □ Feet | □ Face |

□ Swollen lip/tongue

□ Skin rash/ hives/ nettle rash (Please tick all that apply):

| □ Neck | □ Shoulders | □ Back |
| --- | --- | --- |
| □ Chest | □ Upper arms | □ Lower arms |
| □ Abdomen | □ Hands | □ Upper legs |
| □ Lower legs | □ Feet | □ Face |

□ Reaction at the infusion site (such as pain, swelling, hardening)

□ Red eyes

□ Oedema (swelling)

| □ Neck | □ Throat | □ Back |
| --- | --- | --- |
| □ Chest | □ Upper arms | □ Lower arms |
| □ Abdomen | □ Hands | □ Upper legs |
| □ Lower legs | □ Feet | □ Face |

□ Nausea

□ Vomiting

□ Diarrhoea

□ Abdominal pain

□ Stomach pain/ heartburn

□ Coughing

□ Rapid breathing

□ Wheezing

□ Shortness of breath

□ Rapid heart rate

□ Slow heart rate

□ Palpitations

□ Chest pain

□ Irritability

□ Restlessness

□ Dizziness

□ Headache

□ Fatigue

□ Trembling

□ Stinging/ tingling/ numb feeling in one or more limbs

□ Stinging feeling in the mouth/the face

□ Joint pain

□ Muscle spasms/ muscle cramps (Please tick all that apply):

| □ Neck | □ Shoulders | □ Back |
| --- | --- | --- |
| □ Chest | □ Upper arms | □ Lower arms |
| □ Abdomen | □ Hands | □ Upper legs |
| □ Lower legs | □ Feet |  |

□ Muscle pain (Please tick all that apply):

| □ Neck | □ Shoulders | □ Back |
| --- | --- | --- |
| □ Chest | □ Upper arms | □ Lower arms |
| □ Abdomen | □ Hands | □ Upper legs |
| □ Lower legs | □ Feet |  |

□ Don’t know

□ Other, please specify: ________________________________________________________________________________________________________________________________________________

1. ***Which of the following symptoms did you experience within 24 hours of your last infusion? (multiple answers allowed)***

Please tick all options that apply to you

□ None

□ Generally unwell

□ Elevated body temperature (37.5-38.5 C)

□ Fever (temperature >38.5 C)

□ Chills

□ Excessive sweating

□ Pallor

□ Blushing

□ Itching (Please tick all that apply):

| □ Neck | □ Shoulders | □ Back |
| --- | --- | --- |
| □ Chest | □ Upper arms | □ Lower arms |
| □ Abdomen | □ Hands | □ Upper legs |
| □ Lower legs | □ Feet | □ Face |

□ Swollen lip/tongue

□ Skin rash/ hives/ nettle rash (Please tick all that apply):

| □ Neck | □ Shoulders | □ Back |
| --- | --- | --- |
| □ Chest | □ Upper arms | □ Lower arms |
| □ Abdomen | □ Hands | □ Upper legs |
| □ Lower legs | □ Feet | □ Face |

□ Reaction at the infusion site (such as pain, swelling, hardening)

□ Red eyes

□ Oedema (swelling)

| □ Neck | □ Throat | □ Back |
| --- | --- | --- |
| □ Chest | □ Upper arms | □ Lower arms |
| □ Abdomen | □ Hands | □ Upper legs |
| □ Lower legs | □ Feet | □ Face |

□ Nausea

□ Vomiting

□ Diarrhoea

□ Abdominal pain

□ Stomach pain/ heartburn

□ Coughing

□ Rapid breathing

□ Wheezing

□ Shortness of breath

□ Rapid heart rate

□ Slow heart rate

□ Palpitations

□ Chest pain

□ Irritability

□ Restlessness

□ Dizziness

□ Headache

□ Fatigue

□ Trembling

□ Stinging/ tingling/ numb feeling in one or more limbs

□ Stinging feeling in the mouth/the face

□ Joint pain

□ Muscle spasms/ muscle cramps (Please tick all that apply):

| □ Neck | □ Shoulders | □ Back |
| --- | --- | --- |
| □ Chest | □ Upper arms | □ Lower arms |
| □ Abdomen | □ Hands | □ Upper legs |
| □ Lower legs | □ Feet |  |

□ Muscle pain (Please tick all that apply):

| □ Neck | □ Shoulders | □ Back |
| --- | --- | --- |
| □ Chest | □ Upper arms | □ Lower arms |
| □ Abdomen | □ Hands | □ Upper legs |
| □ Lower legs | □ Feet |  |

□ Don’t know

□ Other, please specify: ________________________________________________________________________________________________________________________________________________

1. ***Which of the following symptoms did you experience within 24-48 hours of your last infusion? (multiple answers allowed)***

Please tick all options that apply to you

□ None

□ Generally unwell

□ Elevated body temperature (37.5-38.5 C)

□ Fever (temperature >38.5 C)

□ Chills

□ Excessive sweating

□ Pallor

□ Blushing

□ Itching (Please tick all that apply):

| □ Neck | □ Shoulders | □ Back |
| --- | --- | --- |
| □ Chest | □ Upper arms | □ Lower arms |
| □ Abdomen | □ Hands | □ Upper legs |
| □ Lower legs | □ Feet | □ Face |

□ Swollen lip/tongue

□ Skin rash/ hives/ nettle rash (Please tick all that apply):

| □ Neck | □ Shoulders | □ Back |
| --- | --- | --- |
| □ Chest | □ Upper arms | □ Lower arms |
| □ Abdomen | □ Hands | □ Upper legs |
| □ Lower legs | □ Feet | □ Face |

□ Reaction at the infusion site (such as pain, swelling, hardening)

□ Red eyes

□ Oedema (swelling)

| □ Neck | □ Throat | □ Back |
| --- | --- | --- |
| □ Chest | □ Upper arms | □ Lower arms |
| □ Abdomen | □ Hands | □ Upper legs |
| □ Lower legs | □ Feet | □ Face |

□ Nausea

□ Vomiting

□ Diarrhoea

□ Abdominal pain

□ Stomach pain/ heartburn

□ Coughing

□ Rapid breathing

□ Wheezing

□ Shortness of breath

□ Rapid heart rate

□ Slow heart rate

□ Palpitations

□ Chest pain

□ Irritability

□ Restlessness

□ Dizziness

□ Headache

□ Fatigue

□ Trembling

□ Stinging/ tingling/ numb feeling in one or more limbs

□ Stinging feeling in the mouth/the face

□ Joint pain

□ Muscle spasms/ muscle cramps (Please tick all that apply):

| □ Neck | □ Shoulders | □ Back |
| --- | --- | --- |
| □ Chest | □ Upper arms | □ Lower arms |
| □ Abdomen | □ Hands | □ Upper legs |
| □ Lower legs | □ Feet |  |

□ Muscle pain (Please tick all that apply):

| □ Neck | □ Shoulders | □ Back |
| --- | --- | --- |
| □ Chest | □ Upper arms | □ Lower arms |
| □ Abdomen | □ Hands | □ Upper legs |
| □ Lower legs | □ Feet |  |

□ Don’t know

□ Other, please specify: ________________________________________________________________________________________________________________________________________________

1. ***Do you think the symptoms listed in questions 5 through 7 have anything to do with the infusion?***

□ Yes

□ No

□ Maybe

1. ***Over the past 3 months, I have had an infusion reaction during the following number of infusions:***

□ Never

□ 1 infusion

□ 2 infusions

□ 3 infusions

□ 4 infusions

□ 5 or more infusions

□ Every infusion

If the patient has had at least 1 infusion reaction in the past three months, questions 10 to 13 will appear.

1. ***What has been done in the last 3 months in response to an infusion reaction? (multiple answers allowed)***

□ Infusion stopped completely

□ Infusion paused

□ The infusion speed was adapted

□ A local hospital/ the GP was contacted

□ The Erasmus MC was contacted

□ An acute intervention was needed

□ I was admitted inpatient (hospitalization)

□ Medication was given, if yes, what medication? ________________________________________________________________________________________________________________________________________________

1. ***Did the infusion reaction go away as a result of the intervention mentioned in question 11?***

□ Yes

□ No

□ Don’t know

If the answer option "Infusion paused" is selected at question 10, then question 12 will appear.

1. ***Did the infusion reaction return after restarting the infusion? (If you had multiple infusion reactions, fill in "yes" if the reaction returned at least once after restarting the infusion)***

□ Yes

□ No

□ Don’t know

1. ***After the occurrence of infusion reaction(s), what was done to prevent its recurrence in subsequent infusions? (multiple answers allowed)***

□ One of the infusion steps was adapted

□ The entire infusion schedule was adapted

□ My child receives medication prior to infusion

□ I stopped treatment with enzyme replacement therapy

□ The subsequent infusion was given in the hospital

1. ***Are there any topics that were not discussed in this questionnaire but that you feel are important for us to know?***

______________________________________________________________________________________________________________________________________________________

***Questionnaire adults ≥18 years of age.***

**Prospective questionnaire home infusion project**

General introduction

Thank you for your participation in Erasmus MC's home infusion project. The aim of this project is to gain a better understanding of the patient population receiving home infusions for Pompe disease, whether patients have infusion reactions and how these are managed.

This short questionnaire will ask a number of questions about infusion therapy including what complaints you experienced in the period surrounding the infusion.

We thank you in advance for your cooperation; it is greatly appreciated.

1. ***Have you been ill/have you experienced health complaints prior to administration of the last infusion that could be relevant to safe administration?***

□ Yes, please specify:

______________________________________________________________________________________________________________________________________________________

□ No

□ Don’t know

1. ***In the last two weeks before your last infusion, were you hospitalized/have you visited A&E / the emergency GP?***

□ Yes

□ No

If “Yes” has been answered to question 2, question 3 appears.

1. ***For what reason have you been hospitalized/ visited A&E/ the emergency GP?*** *______________________________________________________________________________________________________________________________________________________*
2. ***Did you experience any health complaints during and/or within 48 hours of your last infusion?***

□ Yes

□ No

If “Yes” has been answered to question 4, questions 5 through 8 will appear.

By means of the following three questions, we would like to find out what symptoms you experienced during your last infusion (question 5), the first 24 hours after your last infusion (question 6) and 24-48 hours after your last infusion (question 7).

1. ***Which of the following complaints did you experience during your last infusion? (multiple answers allowed)***

Please tick all options that apply to you

□ None

□ Generally unwell

□ Elevated body temperature (37.5-38.5 C)

□ Fever (temperature >38.5 C)

□ Chills

□ Excessive sweating

□ Pallor

□ Blushing

□ Itching (Please tick all that apply):

| □ Neck | □ Shoulders | □ Back |
| --- | --- | --- |
| □ Chest | □ Upper arms | □ Lower arms |
| □ Abdomen | □ Hands | □ Upper legs |
| □ Lower legs | □ Feet | □ Face |

□ Swollen lip/tongue

□ Skin rash/ hives/ nettle rash (Please tick all that apply):

| □ Neck | □ Shoulders | □ Back |
| --- | --- | --- |
| □ Chest | □ Upper arms | □ Lower arms |
| □ Abdomen | □ Hands | □ Upper legs |
| □ Lower legs | □ Feet | □ Face |

□ Reaction at the infusion site (such as pain, swelling, hardening)

□ Red eyes

□ Oedema (swelling)

| □ Neck | □ Throat | □ Back |
| --- | --- | --- |
| □ Chest | □ Upper arms | □ Lower arms |
| □ Abdomen | □ Hands | □ Upper legs |
| □ Lower legs | □ Feet | □ Face |

□ Nausea

□ Vomiting

□ Diarrhoea

□ Abdominal pain

□ Stomach pain/ heartburn

□ Coughing

□ Rapid breathing

□ Wheezing

□ Shortness of breath

□ Rapid heart rate

□ Slow heart rate

□ Palpitations

□ Chest pain

□ Irritability

□ Restlessness

□ Dizziness

□ Headache

□ Fatigue

□ Trembling

□ Stinging/ tingling/ numb feeling in one or more limbs

□ Stinging feeling in the mouth/the face

□ Joint pain

□ Muscle spasms/ muscle cramps (Please tick all that apply):

| □ Neck | □ Shoulders | □ Back |
| --- | --- | --- |
| □ Chest | □ Upper arms | □ Lower arms |
| □ Abdomen | □ Hands | □ Upper legs |
| □ Lower legs | □ Feet |  |

□ Muscle pain (Please tick all that apply):

| □ Neck | □ Shoulders | □ Back |
| --- | --- | --- |
| □ Chest | □ Upper arms | □ Lower arms |
| □ Abdomen | □ Hands | □ Upper legs |
| □ Lower legs | □ Feet |  |

□ Don’t know

□ Other, please specify: ________________________________________________________________________________________________________________________________________________

1. ***Which of the following symptoms did you experience within 24 hours of your last infusion? (multiple answers allowed)***

Please tick all options that apply to you

□ None

□ Generally unwell

□ Elevated body temperature (37.5-38.5 C)

□ Fever (temperature >38.5 C)

□ Chills

□ Excessive sweating

□ Pallor

□ Blushing

□ Itching (Please tick all that apply):

| □ Neck | □ Shoulders | □ Back |
| --- | --- | --- |
| □ Chest | □ Upper arms | □ Lower arms |
| □ Abdomen | □ Hands | □ Upper legs |
| □ Lower legs | □ Feet | □ Face |

□ Swollen lip/tongue

□ Skin rash/ hives/ nettle rash (Please tick all that apply):

| □ Neck | □ Shoulders | □ Back |
| --- | --- | --- |
| □ Chest | □ Upper arms | □ Lower arms |
| □ Abdomen | □ Hands | □ Upper legs |
| □ Lower legs | □ Feet | □ Face |

□ Reaction at the infusion site (such as pain, swelling, hardening)

□ Red eyes

□ Oedema (swelling)

| □ Neck | □ Throat | □ Back |
| --- | --- | --- |
| □ Chest | □ Upper arms | □ Lower arms |
| □ Abdomen | □ Hands | □ Upper legs |
| □ Lower legs | □ Feet | □ Face |

□ Nausea

□ Vomiting

□ Diarrhoea

□ Abdominal pain

□ Stomach pain/ heartburn

□ Coughing

□ Rapid breathing

□ Wheezing

□ Shortness of breath

□ Rapid heart rate

□ Slow heart rate

□ Palpitations

□ Chest pain

□ Irritability

□ Restlessness

□ Dizziness

□ Headache

□ Fatigue

□ Trembling

□ Stinging/ tingling/ numb feeling in one or more limbs

□ Stinging feeling in the mouth/the face

□ Joint pain

□ Muscle spasms/ muscle cramps (Please tick all that apply):

| □ Neck | □ Shoulders | □ Back |
| --- | --- | --- |
| □ Chest | □ Upper arms | □ Lower arms |
| □ Abdomen | □ Hands | □ Upper legs |
| □ Lower legs | □ Feet |  |

□ Muscle pain (Please tick all that apply):

| □ Neck | □ Shoulders | □ Back |
| --- | --- | --- |
| □ Chest | □ Upper arms | □ Lower arms |
| □ Abdomen | □ Hands | □ Upper legs |
| □ Lower legs | □ Feet |  |

□ Don’t know

□ Other, please specify: ________________________________________________________________________________________________________________________________________________

1. ***Which of the following symptoms did you experience within 24-48 hours of your last infusion? (multiple answers allowed)***

Please tick all options that apply to you

□ None

□ Generally unwell

□ Elevated body temperature (37.5-38.5 C)

□ Fever (temperature >38.5 C)

□ Chills

□ Excessive sweating

□ Pallor

□ Blushing

□ Itching (Please tick all that apply):

| □ Neck | □ Shoulders | □ Back |
| --- | --- | --- |
| □ Chest | □ Upper arms | □ Lower arms |
| □ Abdomen | □ Hands | □ Upper legs |
| □ Lower legs | □ Feet | □ Face |

□ Swollen lip/tongue

□ Skin rash/ hives/ nettle rash (Please tick all that apply):

| □ Neck | □ Shoulders | □ Back |
| --- | --- | --- |
| □ Chest | □ Upper arms | □ Lower arms |
| □ Abdomen | □ Hands | □ Upper legs |
| □ Lower legs | □ Feet | □ Face |

□ Reaction at the infusion site (such as pain, swelling, hardening)

□ Red eyes

□ Oedema (swelling)

| □ Neck | □ Throat | □ Back |
| --- | --- | --- |
| □ Chest | □ Upper arms | □ Lower arms |
| □ Abdomen | □ Hands | □ Upper legs |
| □ Lower legs | □ Feet | □ Face |

□ Nausea

□ Vomiting

□ Diarrhoea

□ Abdominal pain

□ Stomach pain/ heartburn

□ Coughing

□ Rapid breathing

□ Wheezing

□ Shortness of breath

□ Rapid heart rate

□ Slow heart rate

□ Palpitations

□ Chest pain

□ Irritability

□ Restlessness

□ Dizziness

□ Headache

□ Fatigue

□ Trembling

□ Stinging/ tingling/ numb feeling in one or more limbs

□ Stinging feeling in the mouth/the face

□ Joint pain

□ Muscle spasms/ muscle cramps (Please tick all that apply):

| □ Neck | □ Shoulders | □ Back |
| --- | --- | --- |
| □ Chest | □ Upper arms | □ Lower arms |
| □ Abdomen | □ Hands | □ Upper legs |
| □ Lower legs | □ Feet |  |

□ Muscle pain (Please tick all that apply):

| □ Neck | □ Shoulders | □ Back |
| --- | --- | --- |
| □ Chest | □ Upper arms | □ Lower arms |
| □ Abdomen | □ Hands | □ Upper legs |
| □ Lower legs | □ Feet |  |

□ Don’t know

□ Other, please specify: ________________________________________________________________________________________________________________________________________________

1. ***Do you think the symptoms listed in questions 5 through 7 have anything to do with the infusion?***

□ Yes

□ No

□ Maybe

1. ***Over the past 3 months, I have had an infusion reaction during the following number of infusions:***

□ Never

□ 1 infusion

□ 2 infusions

□ 3 infusions

□ 4 infusions

□ 5 infusions

□ 6 infusions/ Every infusion

If the patient has had at least 1 infusion reaction in the past three months, questions 10 to 13 will appear.

1. ***What actions were taken in the last 3 months as a result of an infusion reaction? (multiple response options possible)***

□ Infusion stopped completely

□ Infusion paused

□ The infusion speed was adapted

□ A local hospital/ the GP was contacted

□ The Erasmus MC was contacted

□ An acute intervention was needed

□ I was admitted inpatient (hospitalization)

□ Medication was given, if yes, what medication? ________________________________________________________________________________________________________________________________________________

1. ***Did the infusion reaction go away as a result of the intervention mentioned in question 11?***

□ Yes

□ No

□ Don’t know

If the answer option "Infusion paused" is selected at question 10, then question 12 will appear.

1. ***Did the infusion reaction return after restarting the infusion? (if you had multiple infusion reactions, fill in "yes" if the reaction returned at least once after restarting the infusion)***

□ Yes

□ No

□ Don’t know

1. ***What actions were taken after the occurrence of the infusion reaction(s) to prevent its recurrence in subsequent infusions? (multiple answers allowed)***

□ One of the infusion steps was adapted

□ The entire infusion schedule was adapted

□ I receive medication prior to infusion

□ I stopped treatment with enzyme replacement therapy

□ The subsequent infusion was given in the hospital

1. ***Are there any topics that were not discussed in this questionnaire but that you feel are important for us to know?***

________________________________________________________________________________________________________________________________________________________
