# Supplementary material for: Home-based enzyme replacement therapy in children and adults with Pompe disease; a prospective study
Source: Orphanet J Rare Dis. 2023 May 8;18:108. doi: 10.1186/s13023-023-02715-4 (PMC10169363; doi:10.1186/s13023-023-02715-4)
Supplement: Supplementary file 3 — Additional file 3. Symptoms occurring during or up to 48 hours after infusion. [file 13023_2023_2715_MOESM3_ESM.docx]

**Additional file 3**

Table S1: Symptoms occurring during or up to 48 hours after infusion

|  | Total | Classic infantile | Atypical infantile | Childhood-onset | Adult |
| --- | --- | --- | --- | --- | --- |
| Complaints during last infusion*, n (% of complaints within group)   - Blushing - Chest pain - Chills - Coughing - Dizziness - Dyspnoea - Fatigue - Headache - Irritability - Itching - Muscle spasm/cramp - Myalgia - Nausea - Generally unwell - Pallor - Palpitations - Paraesthesia - Sweating - Tachycardia - Tachypnoea - Temp 37.5-38.5 - Tingling mouth - Trembling - Urticaria/Skin rash - Other | 2 (3.0%)  2 (3.0%)  4 (6.1%)  1 (1.5%)  1 (1.5%)  2 (3.0%)  12 (18.2%)  6 (9.1%)  2 (3.0%)  2 (3.0%)  1 (1.5%)  2 (3.0%)  2 (3.0%)  6 (9.1%)  3 (4.5%)  2 (3.0%)  1 (1.5%)  1 (1.5%)  1 (1.5%)  1 (1.5%)  3 (4.5%)  1 (1.5%)  1 (1.5%)  3 (4.5%)  4 (6.1%) | 2 (6.5%)  0 (0.0%)  2 (6.5%)  1 (3.2%)  0 (0.0%)  0 (0.0%)  4 (12.9%)  2 (6.5%)  0 (0.0%)  2 (6.5%)  0 (0.0%)  1 (3.2%)  1 (3.2%)  3 (9.7%)  3 (9.7%)  0 (0.0%)  0 (0.0%)  1 (3.2%)  0 (0.0%)  0 (0.0%)  3 (9.7%)  1 (3.2%)  1 (3.2%)  3 (9.7%)  1 (3.2%) | N.A.  N.A.  N.A.  N.A.  N.A.  N.A.  N.A.  N.A.  N.A.  N.A.  N.A.  N.A.  N.A.  N.A.  N.A.  N.A.  N.A.  N.A.  N.A.  N.A.  N.A.  N.A.  N.A.  N.A.  N.A. | 0 (0.0%)  0 (0.0%)  0 (0.0%)  0 (0.0%)  0 (0.0%)  0 (0.0%)  1 (100.0%)  0 (0.0%)  0 (0.0%)  0 (0.0%)  0 (0.0%)  0 (0.0%)  0 (0.0%)  0 (0.0%)  0 (0.0%)  0 (0.0%)  0 (0.0%)  0 (0.0%)  0 (0.0%)  0 (0.0%)  0 (0.0%)  0 (0.0%)  0 (0.0%)  0 (0.0%)  0 (0.0%) | 0 (0.0%)  2 (5.9%)  2 (5.9%)  0 (0.0%)  1 (2.9%)  2 (5.9%)  7 (20.6%)  4 (11.8%)  2 (5.9%)  0 (0.0%)  1 (2.9%)  1 (2.9%)  1 (2.9%)  3 (8.8%)  0 (0.0%)  2 (5.9%)  1 (2.9%)  0 (0.0%)  1 (2.9%)  1 (2.9%)  0 (0.0%)  0 (0.0%)  0 (0.0%)  0 (0.0%)  3 (8.8%) |
| Complaints within the first 24 hours after infusion*, n (% of complaints within group)   - Abdominal pain - Chest pain - Chills - Coughing - Diarrhoea - Dyspnoea - Fatigue - Fever >38.5 - Headache - Heartburn - Irritability - Itching - Muscle spasm/cramp - Myalgia - Nausea - Generally unwell - Pallor - Palpitations - Paraesthesia - Tachycardia - Tachypnoea - Temp 37.5-38.5 - Urticaria/Skin rash - Vomiting - Other | 2 (2.5%)  3 (3.7%)  3 (3.8%)  1 (1.3%)  4 (5.1%)  2 (2.5%)  15 (19.0%)  2 (2.5%)  12 (15.2%)  3 (3.8%)  2 (2.5%)  1 (1.3%)  1 (1.3%)  2 (2.5%)  3 (3.8%)  7 (8.9%)  2 (2.5%)  2 (2.5%)  1 (1.3%)  1 (1.3%)  1 (1.3%)  2 (2.5%)  2 (2.5%)  2 (2.5%)  3 (3.8%) | 2 (4.8%)  1 (2.4%)  1 (2.4%)  1 (2.4%)  2 (4.8%)  0 (0.0%)  8 (19.0%)  2 (4.8%)  6 (14.3%)  1 (2.4%)  0 (0.0%)  1 (2.4%)  0 (0.0%)  1 (2.4%)  2 (4.8%)  5 (11.9%)  2 (4.8%)  0 (0.0%)  0 (0.0%)  0 (0.0%)  0 (0.0%)  2 (4.8%)  2 (4.8%)  2 (4.8%)  1 (2.4%) | N.A.  N.A.  N.A.  N.A.  N.A.  N.A.  N.A.  N.A.  N.A.  N.A.  N.A.  N.A.  N.A.  N.A.  N.A.  N.A.  N.A.  N.A.  N.A.  N.A.  N.A.  N.A.  N.A.  N.A.  N.A. | N.A.  N.A.  N.A.  N.A.  N.A.  N.A.  N.A.  N.A.  N.A.  N.A.  N.A.  N.A.  N.A.  N.A.  N.A.  N.A.  N.A.  N.A.  N.A.  N.A.  N.A.  N.A.  N.A.  N.A.  N.A. | 0 (0.0%)  2 (5.4%)  2 (5.4%)  0 (0.0%)  2 (5.4%)  2 (5.4%)  7 (18.9%)  0 (0.0%)  6 (16.2%)  2 (5.4%)  2 (5.4%)  0 (0.0%)  1 (2.7%)  1 (2.7%)  1 (2.7%)  2 (5.4%)  0 (0.0%)  2 (5.4%)  1 (2.7%)  1 (2.7%)  1 (2.7%)  0 (0.0%)  0 (0.0%)  0 (0.0%)  2 (5.4%) |
| Complaints within 24-48 hours after infusion*, (% of complaints within group)   - Abdominal pain - Blushing - Chills - Coughing - Diarrhoea - Dyspnoea - Fatigue - Fever - Headache - Heartburn - Itching - Myalgia - Nausea - Generally unwell - Pallor - Palpitations - Sweating - Tachycardia - Tachypnoea - Temp 37.5-38.5 - Urticaria/Skin rash - Vomiting - Other | 1 (1.8%)  1 (1.8%)  1 (1.8%)  2 (3.6%) 3 (5.5%)  2 (3.6%)  9 (16.4%)  2 (3.6%)  6 (10.9%)  3 (5.5%)  2 (3.6%)  3 (5.5%)  3 (5.5%)  3 (5.5%)  1 (1.8%)  3 (5.5%)  1 (1.8%)  1 (1.8%)  1 (1.8%)  2 (3.6%)  1 (1.8%)  2 (3.6%)  2 (3.6%) | 1 (3.2%)  1 (3.2%)  0 (0.0%)  2 (6.5%)  2 (6.5%)  0 (0.0%)  5 (16.1%)  2 (6.5%)  3 (9.7%)  1 (3.2%)  2 (6.5%)  1 (3.2%)  2 (6.5%)  1 (3.2%)  1 (3.2%)  0 (0.0%)  1 (3.2%)  0 (0.0%)  0 (0.0%)  2 (6.5%)  1 (3.2%)  2 (6.5%)  1 (3.2%) | N.A.  N.A.  N.A.  N.A.  N.A.  N.A.  N.A.  N.A.  N.A.  N.A.  N.A.  N.A.  N.A.  N.A.  N.A.  N.A.  N.A.  N.A.  N.A.  N.A.  N.A.  N.A.  N.A. | N.A.  N.A.  N.A.  N.A.  N.A.  N.A.  N.A.  N.A.  N.A.  N.A.  N.A.  N.A.  N.A.  N.A.  N.A.  N.A.  N.A.  N.A.  N.A.  N.A.  N.A.  N.A.  N.A. | 0 (0.0%)  0 (0.0%)  1 (4.2%)  0 (0.0%)  1 (4.2%)  2 (8.3%)  4 (16.7%)  0 (0.0%)  3 (12.5%)  2 (8.3%)  0 (0.0%)  2 (8.3%)  1 (4.2%)  2 (8.3%)  0 (0.0%)  3 (12.5%)  0 (0.0%)  1 (4.2%)  1 (4.2%)  0 (0.0%)  0 (0.0%)  0 (0.0%)  1 (4.2%) |

N.A.= Not applicable * Question was only available if the patient stated to have experienced any health complaints during and or within 48 hours from last infusion.
